# Cotton Dust Exposure and Respiratory Disorders among Textile Workers at a Textile Company in the Southern Part of Benin

**DOI:** 10.3390/ijerph13090895

**Published:** 2016-09-08

**Authors:** Antoine Vikkey Hinson, Virgil K. Lokossou, Vivi Schlünssen, Gildas Agodokpessi, Torben Sigsgaard, Benjamin Fayomi

**Affiliations:** 1 Unit of Teaching and Research in Occupational Health and Environment, Faculty of Sciences of the Health, University of Abomey-Calavi, Abomey-Calavi, 01 PO 188 Cotonou, Benin; lovik83@yahoo.fr (V.K.L.); bfayomi2@yahoo.fr (B.F.); 2 Section for Environment, Occupation and Health, Department of Public Health, Aarhus University Denmark, Nordre Ringgade 1, 8000 Aarhus C, Denmark; vs@ph.au.dk (V.S.); ts@ph.au.dk (T.S.); 3 Unité D’enseignement et de Recherche en Pneumo-Phtisiologie, Université d’Abomey-Calavi, Abomey Calavi, 01 PO 321 Cotonou, Benin; aggildas@yahoo.fr

**Keywords:** respiratory disorders, cotton dust, byssinosis, FEV_1_, FVC, Benin

## Abstract

The textile industry sector occupies a prominent place in the economy of Benin. It exposes workers to several occupational risks, including exposure to cotton dust. To assess the effect of exposure to cotton dust on the health of workers, this study was initiated and conducted in a Beninese cotton industry company. The objective of the study was to evaluate the respiratory disorders among the textile workers exposed to cotton dust and the cross-sectional study involved 656 subjects exposed to cotton dust and 113 non-exposed subjects. The methods used are mainly based on a survey using a questionnaire of organic dust designed by the International Commission of Occupational Health (ICOH); and on the measures of lung function parameters (FEV_1_ and FVC). The main results of the different analyzes revealed that subjects exposed to cotton dust have more respiratory symptoms than unexposed subjects (36.9% vs. 21.2%). The prevalence of chronic cough, expectorations, dyspnoea, asthma and chronic bronchitis are 16.8%, 9.8%, 17.3%, 2.6%, and 5.9% respectively among the exposed versus 2.6%, 0.8%, 16.8%, 0% and 0.8% among the unexposed subjects. The prevalence of byssinosis is 44.01%.The prevalence of symptoms is dependent on the sector of activity and the age of the subject. These results should encourage medical interventions and technical prevention especially since the textile industry occupies an important place in the Benin’s economy.

## 1. Introduction

Byssinosisis a chronic respiratory disease that is seen among workers exposed to cotton, flax, and soft hemp dust [1]. Also called “Monday morning dyspnea”, byssinosis is a disease present in workers exposed to cotton dusts and characterized by respiratory symptoms both histologically and physiologically, with decline of the respiratory function [2]. Although in the industrialized world, there has been a significant decline in the prevalence of cotton dust lung diseases, studies show an increasing incidence in the developing world. With rapid industrialization of the developing world, cotton dust-induced lung diseases are poised to become a global health problem [3]. Prevalences elsewhere are as follows: Turkey in 2002: 14.2%; Indonesia: 30%; In Pakistan: 35.6% (2008) and 10.5% (2013) [4,5,6,7]. In developing countries, notably in Africa, the cotton industry occupies an important place. The cotton sector, also called “white gold”, is expanding considering the size of cotton production and the number of people employed in this sector. The prevalence of byssinosis in Africa was as follows: in Sudan 42%; in Ethiopia, 43% (1991) and 44% (1994); in Benin for grade 3, 21.1% (2014) at the north part of the country and working half time/year [8,9,10,11].

The Republic of Benin, a West African country, has low incomes and does not escape the predominance of the “white gold” in its economy [12]. Indeed in Benin, agriculture is the main activity, occupying more than 70% of the working population and representing about 40% of the gross domestic product [13]. The cotton sector is the main economic motor and very important in all activity sectors [12]. Exposure to cotton can cause lung diseases with considerable socio-sanitary consequences [14]. The textile industries sector exposes workers to several respiratory occupational diseases, of which byssinosis is the most important [15]. However there is a scarcity of data showing these kinds of health issues in Benin and in other West-African countries. It is crucial to highlight byssinosis because in companies like the Benin Textile Company some employees are exposed for up to 12 h a day to a high level of dust unlike the factory in the north where the workers were active for only 6 months of the year due to economic problems where research showed a byssinosis prevalence in 2014 of 21.1% at grade3 among 109 cotton dust exposed workers [11]. All this raises the question: what then is byssinosis prevalence in Benin? In response, the aim of this study was therefore to evaluate the respiratory disorders (mainly byssinosis) among textile workers exposed to cotton dust within a company, and furthermore to assess the consequences of these respiratory symptoms.

## 2. Materials and Methods

### 2.1. Study Population

The frame of this study is the Beninese Company of Textile (CBT) which is situated at the southern part of the country in Mono Department. The factory was established in October 2002 with 51% Chinese and 49% Benin ownership. The total workforce in the factory numbers 1316.

### 2.2. Description of the Activities in the Cotton Factory

Only cotton is manufactured in the factory, which has two sections: administrative and technical. The administrative section employees who work 8 h/day (8:00–12:30 and 15:00–18:30). The technical section is subdivided into three departments as follows: the spinning department, the weaving department and the general service department. The general service department deals with maintenance of the factory as a whole. The employees of the technical department (beside the general service which works as administrative direction), alternate in three shifts (8 h/shift) to ensure 24 h continuous production each day.

- The *Spinning Department* makes cotton into threads through the *Preparation Section* (threshing, milling, stretching and cross-pinning) and spinning section.
- The *Preparation* and *Small Preparation* sections. The large preparation extends from threshing to milling. During threshing, the cotton balls are opened by peeling machines which at the same time eliminate dirt and dust to help produce a mixture of the various fibres. Finally, the thresher helps complete the operations of braiding by clearly separating the fibres and by making the rollers of cotton tablecloths. The milling section carries out the final operation of purification by transforming the small tufts of cotton into well separated and open fibers of cotton. The small preparation deals with stretching and cross-pinning. Stretching is achieved with appropriate machines and ensures the “parallelism” of the ribbons coming from milling by softening them. At the cross-pinning level, the stretched flexible ribbons are processed into locks of cotton.
- The *Spinning Department* uses machines of a “proceed on to spinning” type. Spinning helps to produce cotton threads of required size in two ways from the locks of cotton: the chain thread and the weft thread. The weft threads go directly to the weaving whereas the chain threads undergo other processing.
- The *Weaving Department* is divided into two major parts: pre-weaving and weaving. Pre-weaving comprises four different stages. Firstly winding, which consists in unrolling the chain threads to roll them up on large spools thanks to the winding machine. Then, warping where a station is equipped with wrappers and the threads are rolled up on a cylinder called a “beam”. Third comes the gluing stage by means of gluers that pass the threads coming from the beams through glue in order to increase their resistance to rubbing. The glued threads are then dried before being rolled up in new beams. The final stage, drawing-in, involves preparing the previously glued threads for weaving by passing the glued beam threads in the gills and the combs. Weaving consists of two stages: weaving itself and verification. Cloth is obtained from the chain threads placed vertically and the weft threads placed horizontally. Cloths obtained through weaving are conveyed to the verification chamber where they are examined and folded in case they are not properly woven. Cloths are afterwards stocked in the warehouse.

### 2.3. Selection of Participants

- *Inclusion criteria*: All workers having at least 2 years of experience in the production chain were included in the exposed population. The population unexposed to cotton dusts included both the general administration staff members of the company and external workers in the informal sector located in the surroundings of the company having at least 2 years of job activities. Those who did not meet the inclusion criteria were excluded.
- The *sample size* was calculated using Schwartz formula: *N* = ε^2^*p* (1 − *p*)/*i*^2^. The prevalence of byssinosis in Africa is on average 40% according to the previous works by Karim [7] and by Woldeyohannes [9]. The minimal size of the sample of the exposed people with a margin of error of 5% was 370 subjects. The unexposed sample size was estimated to include 1/3 of the minimal size of the exposed people: a total of 123 unexposed persons.
- *Sampling method*: Through a random sampling, 370 exposed persons with at least 2 years employment at the factory out of 1208 employees were selected. For the unexposed persons, all management staff members who accepted were included and also people in the neighborhood of the company with at least 2 years employment in the factory. Taking into account the possibilities of incomplete filling in of the questionnaires or the existence of invalid questionnaires, the size of the sample was overestimated during the period of data collection, so 769 individuals were then contacted.
- *Study design*: A cross-sectional study with a control group was carried out from January to April 2013.

### 2.4. Data Collection

During an interview, the participants were asked to answer questions about respiratory symptoms taken from the International Commission on Occupational Health (ICOH) questionnaire. The questionnaire included symptoms such as cough, phlegm, chest tightness, feverishness, wheeze, breathlessness, and sneezing and itchy eyes, as well as any family history of allergy. The questionnaire also included smoking habits for recording of a full smoking history. A complete occupational history was also obtained.

Chronic bronchitis was diagnosed in accordance with the definitions from the British Medical Research Council [16]. The persons with the same criteria but without phlegm were diagnosed as having a chronic dry cough. The diagnosis of byssinosis was based on “WHO grading system for byssinosis” mainly on lung function classification: no effect: FEV_1_ = 80% of predicted value; mild to moderate effect FEV_1_ 60%–79% of predicted value; severe effect: FEV_1_ less than 60% of predicted value [17]; and also on the Schilling criteria [18]: Grade 0: No symptoms of chest tightness or breathlessness on Monday; grade ½ occasionally chest tightness on the first day of the working week; grade 1: chest tightness on every first day on the working week; grade 2: chest tightness on the first and more days of the working week; and grade 3: grade 2 plus chronic lung impairment: FEV_1_ < 80% of the predicted value. The persons who suffered from work-related respiratory problems without symptoms occurring the first workday were diagnosed as having a non-specific respiratory problem. Asthma was diagnosed if the person experienced wheeze and dyspnoea, linked either with exposure to a well-known allergen or with a non-specific agent.

### 2.5. The Pulmonary Function Test

The pulmonary functions were tested in the workers before and after the shift. The pulmonary function test was done with a dry spirometer (Vitalograph, Buckingham, UK). Before entering the workshop, forced expiratory volume in one second (FEV_1_) and forced expiratory capacity (FVC) of each worker were recorded in accordance with the American Thoracic Society (ATS) guidelines [19]. As all the employees of the production chain of the factory compulsorily worked once in their shift/day, it was not possible for them to have a break before resuming duty. The test was repeated at the end of the shift after two hours of rest. Only the best of three tests before and after work, respectively, was retained. As most of the unexposed workers were itinerant and nomadic it was difficult to repeat the tests for them. The FEV_1_ predictive values were calculated using standard equations taking into account sex, age, race and weight. The FEV_1_ values pre-shift and post-shift were used to calculate the percentage change during the day. The FEV_1_ predictive value have been calculated with the following formulas:

FEV_1_ for Africans = 0.88 × ((0.0414 × Ht) − (0.0244 × age) − 2.19) in the male (Lap et al. [20]).
FEV_1_ for Africans = 0.88 × ((0.0342 × Ht) − (0.0255 × age) − 1.578) in the female (Lap et al. [20]).

The FEV_1_ post-shift values were subtracted from the pre-shift values, and negative differences indicate a decrease in pulmonary function [2]. This difference of volume is divided by the highest maximal respiratory volume obtained from the worker (FEV_1_ max) during the period of work in order to obtain the variations in terms of percentage of the pulmonary function (%FEV_1_ changes) [2].

### 2.6. Data Analysis

Data entry was performed using the Access 2007 software (Microsoft, Redmond, WA, USA). The data was exported to Microsoft Excel 2007 and the Statistical Package for Social Sciences software (SPSS version 17 for Windows, SPSS Inc., IBM, Armonk, NY, USA). After summary statistical comparisons were made with analysis of the variance and with Students *t* test. For categorical values the Pearson’s chi-square test was used. A *p* value < 0.05 was considered statistically significant. In multivariate analyses, we firstly evaluated the association between cotton dust exposure and byssinosis symptoms using logistic regression and secondly we evaluated the association between cotton dust exposure and FEV_1_ using linear regression. In the two models, others co-variables are: age, sex and smoking.

### 2.7. Ethical Consideration

This study received the agreement of the management of Benin Textile Company to conduct the study by the agreement letter ref: CBT/DAF/SRH/N112/2013/mcrt LOKOSSA. Prior to any inclusion, participants gave informed consents to participate in the study. Throughout the study participants were allowed to voluntarily withdraw if they wished.

## 3. Results

### 3.1. Socio-Demographic Characteristics of the Sample

Of 769 subjects approached, all 769 completed the symptom questionnaire. 185 completed pre-shift spirometry, and 394 completed post-shift spirometry. The average ages of participants were 29.07 ± 6.13, and 745 were men. Notably, only 12 out of the 769 participants were smokers. When evaluating the baseline characteristics of the exposed vs. unexposed groups (Table 1), the exposed participants tended to be younger, taller, and thinner (*p* < 0.05).

### 3.2. Respiratory Symptoms among Participants

There are more signs of respiratory tract irritation in those exposed to cotton dust than unexposed (Table 2): (*p* < 0.05).

### 3.3. Prevalence of Byssinosis

When evaluating the prevalence of byssinosis in this population, we found that those not directly exposed to cotton dust had a lower likelihood of symptoms of byssinosis (as defined by the Schilling criteria (Table 3).

When looking at each grade of byssinosis, there were no statistically significant differences between groups, although the numbers were small (*p* = 0.68). The number of exposed workers for grade 1/2 to 3, were always higher than the unexposed. When evaluating chronic byssinosis as defined by WHO criteria for byssinosis using lung function testing, we found that when evaluating post-shift FEV_1_, those directly exposed to cotton dust had significantly lower FEV_1_ (Table 4).

The subjects presenting a moderate deficit represent 33% proportion while the people having a severe deficit are approximately 11% of the sample so in total the proportion was 44%.

### 3.4. Influencing Factors of Byssinosis

In further univariate analyses (Table 5), we further explored the effects of age, seniority (based on years worked), smoking, and educational level on FEV_1_.

The prevalence of byssinosis (lung function decline) is higher at the spinning and weaving departments than in the general service and in the administration department. The spinning and weaving departments are obviously the sectors in which there are an intensive exposure to cotton dust (*p* < 0.05).

### 3.5. Impacts of the Co Factors

In multivariate analyses, we evaluated the association between cotton dust exposure and both byssinosis symptoms and FEV_1_ (Table 6 and Table 7).

This study used a logistic regression to see the effect of smoking and exposure to cotton dust on the presence of byssinosis symptoms with factors of adjustment, the age and the sex.

Smoking multiplies by 6 (OR = 11.16 [2.96; 42.01]) the risk of having byssinosis symptoms. The exposure to cotton dust had no statistically significant influence on the respiratory symptoms.

This study used the linear regression to see the effect of smoking and exposure to cotton dust on the FEV_1_ with factor adjustment the age and the sex.

The smoking and exposed to cotton dust have no statistically significant influence on FEV_1_ (*p* = 0.82 and *p* = 0.76). The gender variable was excluded because it was not efficient in the model.

## 4. Discussion

### 4.1. Respiratory Symptoms

All individuals presenting at least one symptom are considered in this study as symptomatic. Our study showed that there is no difference between exposed and unexposed workers concerning the prevalence of respiratory symptoms. This result is not in accordance with the one obtained by Laraqui and his collaborators who got a prevalence ratio of 2.46 (45.1% in the exposed individuals against 16.3% in the unexposed individuals) [21]. When we evaluated the prevalence of symptoms in this cohort, comparing individuals directly exposed to those indirectly exposed to cotton dust, there was no difference between the exposed group and the unexposed (*p* > 0.05), but when we explored in detail the respiratory symptoms, we noted increased cough, sputum production, chest tightness, and dyspnea, with 10 (1.53%) reporting symptoms consistent with asthma and 22 (3.36%) reporting symptoms consistent with chronic bronchitis. This lack of difference of respiratory symptoms could be explained by memory biases during the interviews. Remarkably, even those not directly exposed to cotton dust had a high proportion of respiratory symptoms, and 21.24% reported some form of respiratory symptoms hence revealing exposure to cotton dust as a public health problem. Among the symptoms declared by the interviewed workers, the breathing discomfort symptoms were statistically less frequent in the individuals exposed to cotton dusts (9.9%) than in the unexposed individuals (16.82%). This result does not corroborate the one obtained by Doker et al. during a study of 350 workers from 20 factories working in the cotton sector with a prevalence recorded of 34.5% [22]. A similar result has also been obtained by Zuskin et al. [23] at the end of a study with 400 workers exposed and 138 non-exposed. They noted a higher prevalence of the chronic breathing discomfort respiratory symptoms in the exposed individuals than in the non-exposed, with a significant difference. Even the unexposed workers reported respiratory symptoms. This further demonstrates the important public health significance of cotton dust exposure in Benin. Not only will the workers directly working with cotton be affected, but also those working in administration and even neighbors.

Breathing discomfort symptoms were more frequent in sectors known to generate high dust levels such as spinning (41.5%) and weaving (34%). This report is confirmed as daCosta and his collaborators noted a higher frequency (28.3%) of breathing discomfort symptoms in the workers in cotton spinning zones, notably those at work in the initial phases of the spinning process (opening of cotton bales and carding) against 12.7% in the weaving zones [24]. This respiratory symptomatology combines a large number of symptoms; the most frequent of which are chronic cough, expectoration, chest constriction and dyspnoea.

### 4.2. Chronic Cough

Chronic cough is defined in this study as a cough evolving for at least three months. In our study the prevalence of cough was 16.8% and was more frequent in the exposed individuals (16.8%) than in the non-exposed individuals (2.9%): *p* < 1‰. This result is in accordance with that of Ahassan et al. in their study on professional exposition and breathing diseases in the workers of textile sector in developing countries [25]. During this study, those authors obtained a cough rate of 42.9% and a strong relation between the existence of chronic cough and professional practice in a dusty environment. In a more recent study, Nafees et al. recorded in Pakistan a prevalence of 7.5% for chronic cough [7].

### 4.3. Bronchial Expectorations

The prevalence of bronchial expectoration was 7.9% in our study and more often present in the exposed individuals (9.8%) than in individuals not exposed to dusts (0.8%), i.e., a substantial difference. Our results are in accordance with those published by Nafees and collaborators who observed a prevalence rate of about 12.9% in Karachi [7]. Besides, Laraqui and his collaborators recorded in Morocco a prevalence rate of 18.42% [21].

### 4.4. Chest Constriction and Breathing Discomfort

Our study pointed out some respectively prevalence rates of 7.2% for chest constriction and 9.9% for breathing discomfort. It is important to note that those two symptoms present higher prevalence in the exposed workers than unexposed. Our results are in accordance with those of Zuskin and his collaborators [23]. Indeed, in their comparative study, these authors too recorded a higher prevalence in the exposed individuals with a more considerable difference for breathing discomfort. Our results are also in accordance with those of Laraqui and his collaborators in Morocco who recorded a rate of 13.4% for dyspnoea [21].

### 4.5. Chronic Bronchitis

Chronic bronchitis has been defined in this study by the existence of a cough accompanied by a regular expectoration of at least 3 months in individuals. It appeared in our study more frequently in the exposed individuals (3.4%) than in non-exposed individuals (0.9%). Our chronic bronchitis prevalence was close to the 5.3% recorded by Glindmeyer [26]. The scientific literature reveals varied prevalence rates of chronic bronchitis ranging from 5.7% [18] to 10.9% [26] through 6% [27] in exposed individuals. Those rates vary according to age and time of exposure. The report of high prevalence of chronic bronchitis in the group of exposed individuals has also been shown by Massins and his collaborators who observed prevalence rates of 4.7% respectively in the exposed workers and 1.7% in the non-exposed individuals (*p* < 0.01) [28]. Similar results have been obtained by Akrout et al. [29]. Those authors comparing workers exposed to cotton dusts and other non-exposed workers noted a statistically higher prevalence chronic bronchitis in the group of exposed workers (18.33%) than in the group of the non-exposed individuals (5.6%). Finally, in a study concerning 2991 textile workers, the authors noted that the prevalence of chronic bronchitis is high in cotton workers [1].

### 4.6. Respiratory Functional Explorations

The parameters of the respiratory function which have been observed within this study are the FEV_1_ and the FVC. This study enabled us to understand that there is a more important decrease of the FEV_1_ in the exposed individuals than in non-exposed individuals. Many studies also established this relation between exposition to textile dusts and anomalies of the respiratory function. Indeed, an important reduction of the FVC and the FEV_1_ has also been recorded by Fishwick et al. [30], Jiang et al. [31], Love et al. [32] and Niven et al. [33]. A longitudinal study led by Ma et al. in 60 new employees of the textile industry observed over 5 years showed that the FEV_1_ decreases with the length of exposure to cotton dusts [34]. Christiani et al., in another longitudinal study over 11 years with 445 cotton workers, measuring the respiratory function and the exposureto cotton dusts, also noted a more important decrease of the FEV_1_ in exposed individuals [35]. Akrout et al. also recorded a reduction of the respiratory parameters in the group exposed to cotton in comparison with the non-exposed individuals [24]. In general, several studies agree on the fact that the level of the respiratory functions decreases depends on the concentration of dust in the workplace, on the time of exposition, on age and on tobacco addiction [9,28,31,36,37]. Finally, it is important to note that respiratory functional disorders reported in this study are generally slight. This can be explained by the healthy worker effect Workers with an important respiratory anomaly may abandon their job.

### 4.7. Prevalence of Byssinosis

Byssinosis was for a long time the subject of misunderstanding and controversies. First of all in the 1950s, the word “byssinosis” described acute symptoms solely due to cotton hemp and linen dust exposure (Monday symptoms) [14]. Then the term came to combine acute respiratory and chronic symptoms (obstructive affection; fixed and durable). According to the WHO grading system for byssinosis [17], the prevalence of lung function chronic change from mild to severe effect in our study is 44%. These results according to the Schilling criteria tend to imply that there is not an important difference between the prevalence of byssinosis as observed in individuals exposed to cotton dusts and the non-exposed individuals, but if we combined grades 1/2 to 3 into one category and compare the exposed to unexposed, we will find a significant difference. Already the number of exposed workers for grades 1 to 3 is higher than the unexposed. We do take into account the subjective characteristic of the outbreak of symptoms showing the existence of byssinosis (recall problems and the application of the questionnaire) and considering the fact that the workers of the Company do not have rest days nor holidays. Our prevalence figure is close to the one observed in the United States by Bouhuys which is 38% [38]. It is important to note that initially in developed countries some very high prevalence rates were recorded: 63% by Schilling for example [18].

Those figures decreased subsequently with Newman-Taylor recording a rate of 10% in the carders against 3% in the spinners [39]. The current prevalence of the disease varies according to studies and is roughly 1.5% [40], 3.5% [26,36], of 6% [9,24], of 10% [22,41] and 59% [41]. Today, whereas the prevalence rates decrease in the developed countries, they are still very high in developing countries.

The distribution of these prevalence rates can be explained in several ways. Firstly, the studied populations do not come from the same workplaces or departments (large preparation, spinning, carding, salaried employees of a company, etc.). Secondly the definition of byssinosis used is not always the same from one study to another and can include different stages. Also the different conditions of exposure to cotton dusts as well as the modes of interviewing the individuals can constitute additional reasons for difference in prevalence rates recorded.

This study shows that the prevalence of byssinosis is considerably higher in the exposed individuals (44.01%) than in the non-exposed workers (13%). The prevalence of byssinosis, as our study shows, varies according to department with the prevalence of byssinosis highest higher in spinning and weaving workers than in employees of other departments. A similar result exists for Egypt, where the prevalence of byssinosis varied from 13% in the carders (carding and beating), 21% in the openers (spinning) to 0% in the enrollers [36]. The situation is also very similar in Ethiopia where the prevalence of byssinosis varies from 38% in the carders to 17% in the enrollers and to 4% in the weavers [9]. In addition the age of the workers also constitutes a key risk factor for byssinosis as the prevalence of byssinosis increases with age and length of time in the industry.

Tobacco consumption didnot influence the prevalence of byssinosis in our study. This could be due to the small number of smokers we found and Akrout’s study found tobacco to be an important factor in the genesis of byssinosis [38]. Schachter also found tobacco an important factor in the prevalence of morbidities in textile workers who smoked in contrast with their non-smoking colleagues [42]. Finally, the scientific literature shows that the prevalence of byssinosis varies according to the type of cotton used (raw, coarse, middle or thin), tobacco consumption, the level of dust at the workshops and professional seniority [21].

## 5. Conclusions

The textile industrial sector exposes the workers to various occupational hazards of which one is the exposure to cotton dusts. Our study allowed us to estimate the prevalence of respiratory disease and symptoms including byssinosis and abnormal lung function. The subjects exposed to cotton dust present more respiratory symptoms than the unexposed subjects. The prevalence of byssinosis in the Beninese Company of Textiles was 44.01%. This prevalence varies in the company according to the age, and to sectors of activities of the subject. Even the unexposed workers also have byssinosis symptoms. That demonstrates the public health impact of cotton dust exposure in Benin. It is not just the workers directly working with cotton who are affected, it is those working in administration and even neighbors who are affected.

This study has not been able to include samplings of the atmosphere to measure the level of dust in the different work sectors. That aspect will be addressed in the second part of the study. These results should lead to prioritizing medical interventions and technical prevention especially as the textile industry occupies such a dominant place in the economies of developing countries.

### 5.1. Limitations of the Study

During this study, many difficulties and limits were encountered and should be flagged. Concerning information biases, the results of the questionnaire were obtained through direct interviewing. This method could contain some biases in the delivery of information as it is based on the patients “memory and declaration”. The absence of a rest period for the workers because of the work organization including not even at weekends (all the workers must work every day), made it difficult to appreciate of the definition of byssinosis as the “Monday dyspnoea”. Almost all spirometry was completed post-shift and this was a significant limitation. The diagnosis of asthma is problematic as it seems to be based on self-reporting. The lack of exposure assessment (cotton dust or endotoxin levels) was also a limitation. This last aspect will be the object of follow-up in the second part of the study in the coming months.

### 5.2. The Strengths of the Study

This is the first study from West Africa on the effects of cotton dust exposure on health. Other strengths include large number of participants, inclusion of both symptoms and lung function testing, low proportion of smokers in our study population, and presence of a “control group”.

## Figures and Tables

**Table 1:** General characteristics of the sample.

Characteristics	Exposed (*n* = 656)	Unexposed (*n* = 113)	*p*
Age (year)	28.6 ± 5.6	31.9 ± 8	0.0001
Height (cm)	169.3 ± 7	165.4 ± 7.3	0.0001
Weight (kg)	66.2 ± 9.5	68.6 ± 14.1	0.06
Seniority (year)	6.3 ± 2.7	6.1 ± 0.6	0.05
Smoking (n)	9	3	0.56
Male N (%)	656 (85.30)	89 (11.57)	0.0001 *
Baseline FEV_1_ (L)	3.00 ± 0.70	3.04 ± 0.54	0.79
% Predictive value FEV_1_	74.12%	77.28%	0.82
Baseline FVC (L)	3.69 ± 0.96	3.64 ± 1.42	0.79
% Predictive value FVC	130.92%	132.73%	0.77

* = fisher exact test.

**Table 2:** Prevalence of symptoms and affections according to the exposure to cotton dusts.

Symptoms/Diseases	Exposed (n/N%)	Unexposed (n/N%)	*p*
Respiratory (*N* = 769)	139/656 (21.1%)	24/113 (21.2%)	0.9
**Signs of the respiratory tract irritation**			
Cough (*N* = 481)	63/376 (16.8%)	3/105 (2.9%)	0.001
Bronchial secretions (*N* = 769)	37/656 (5.6%)	1/113 (0.9%)	0.003
Chest constriction (*N* = 769)	47/656 (7.2%)	3/113 (2.7%)	0.003
Dyspnoea (*N* = 769)	65/656 (9.9%)	19/113 (16.8%)	0.05
Asthma (477)	10/375 (1.5%)	0/102) (0%)	0.088
Chronic bronchitis (769)	22/656 (3.4%)	1/113 (0.9%)	0.001

**Table 3:** Gradation of the byssinosis according to schilling criteria.

Byssinosis Grade	Exposed	Unexposed	Total	*p*
Grade 0	517	89	606	
Grade 1/2	53	9	62	
Grade 1	41	11	52	0.68
Grade 2	4	0	4	
Grade 3	9	2	11	
Total	624	111	735	

**Table 4:** Chronic lung function changes according to WHO grading system for byssinosis.

Chronic Anomalies	Exposed (*n* = 393)	Unexposed (25)	*p*
No effect (FEV_1_ ≥ 80%) (n, %)	220 (56%)	19 (76%)	0.001
Mild to Moderate effect (FEV_1_ 60%–79%) (n, %)	130 (33.06%)	5 (20%)	0.001
Severe effect (FEV_1_ less than 60%) (n, %)	43 (10.94%)	1 (4%)	0.001

**Table 5:** Prevalence of byssinosis (lung function decline) according to the socio-demographic and socio-professional characteristics.

Prevalence of Byssinosis	Subjects with FEV_1_ < 80% *n* = 179	Subjects with FEV_1_ > 80% *n* = 239	*p*
**Sector of activities**			0.016
Spinning	31 (46.3%)	59 (39.9%)	
Weaving	21 (31.3%)	39 (26.4%)	
General service	11 (16.4%)	15 (10.1%)	
Administration + informal sector	4 (6.0%)	35 (23.7%)	
**Age (years)**			0.05
<20	4 (2.2%)	10 (4%)	
20–25	50 (27.9%)	44 (17.5%)	
25–30	70 (39.1%)	80 (31.9%)	
30–35	38 (21.2%)	73 (29.1%)	
>35	17 (9.5%)	44 (17.5%)	
**Years worked (years)**			0.35
2–5	15 (8.38%)	15 (6%)	
5–10	150 (83.8%)	208 (82.9%)	
>10	14 (7.8%)	28 (11.1%)	
**Smoking**			0.557
Non-smokers	1 (11.1%)	8 (88.9%)	
Smokers	1 (33%)	2 (67%)	
**Level of instruction**			0.514
Primary	24 (47.1%)	44 (44.5%)	
Secondary 1	16 (31.4%)	38 (38.4%)	
Secondary 2	7 (13.7%)	7 (7.1%)	
University	4 (7.8%)	10 (10.1%)	

**Table 6:** Association of byssinosis symptoms with cotton dust exposure.

Factors		OR	95% of CI	*p*-Value
**Exposure to cotton dust**	No			
	Yes	1.19	[0.67; 2.10]	0.56
**Smoking**	No			
	Yes	11.16	[2.96; 42.01]	<0.001
**Gender**	Female			
	Male	0.87	[0.28; 2.67]	0.81
**Age**		1.03	[1.00; 1.06]	0.05

**Table 7:** Association of FEV_1_ with cotton dust exposure.

Factors		Coef	95% of CI	*p*-Value
**Exposure to cotton dust**	No			
	Yes	−0.16	[−1.23; 0.90]	0.76
**Smoking**	No			
	Yes	−0.17	[−1.66; 1.33]	0.82
**Age**		−0.003	[−0.02; 0.02]	0.78
